# Metformin suppresses Nrf2-mediated chemoresistance in hepatocellular carcinoma cells by increasing glycolysis

**DOI:** 10.18632/aging.103777

**Published:** 2020-09-14

**Authors:** Liangyu Cai, Xin Jin, Jiannan Zhang, Le Li, Jinfeng Zhao

**Affiliations:** 1 Department of Hepatobiliary and Pancreatic Surgery, Key Laboratory of Nanobiological Technology of Chinese Ministry of Health, Xiangya Hospital, Central South University, Changsha 410008, Hunan, China; 2 Wuxi TCM Hospital Affiliated to Nanjing University of Chinese Medicine, Wuxi 214071, Jiangsu, China; 3 Hunan Yuantai Biotechnology Co., Ltd, Changsha 410000, Hunan, China

**Keywords:** liver cancer, HepG2, metformin, Nrf2, glycolysis

## Abstract

The diabetes drug metformin has recently been shown to possess anti-cancer properties when used with other chemotherapeutic drugs. However, detailed mechanisms by which metformin improves cancer treatment are poorly understood. Here we provide evidence in HepG2 hepatocellular carcinoma cells that metformin sensitizes cisplatin-resistant HepG2 cells (HepG2/DDP) through increasing cellular glycolysis and suppressing Nrf2-dependent transcription. We show that metformin increases glucose uptake and enhances glucose metabolism through glycolytic pathway, resulting in elevated concentrations of intracellular NADPH and lactate. Consistently, high glucose medium suppresses Nrf2-dependent transcription and sensitizes HepG2/DDP cells to cisplatin. Elevated glycolysis was required for metformin to regulate Nrf2-dependent transcription and cisplatin sensitivity, as inhibition of glycolysis with 2-Deoxy-D-glucose (2-DG) significantly mitigates the beneficial effect of metformin. Together, our study has revealed an important biological process and gene transcriptional program underlying the beneficial effect of metformin on reducing chemo-resistance in HepG2 cells and provided new information on improving chemotherapy of liver cancers.

## INTRODUCTION

Hepatocellular carcinoma (HCC) is one of the most common type of primary liver cancers, which accounts for roughly 70% of all liver cancers [1, 2]. In the United States for example, HCC ranks first among newly diagnosed cancers and is the fastest rising cause of cancer-related deaths [2]. East Asia, Southeast Asia and Sub-Saharan Africa have the highest liver cancer incidence, likely due to food contaminations from the fungi toxin aflatoxin [3, 4]. HepG2 is an immortalized liver carcinoma cell line derived from a Caucasian male patient with HCC [5, 6]. HepG2 cells have been widely used to investigate the biology and treatment of HCC, such as tumorigenesis signaling [7], drug resistance [8] and immunotherapy [9]. Like many other cancer cells, HepG2 often develops multiple drug resistance phenotype after chemotherapy. Continuous culturing of HepG2 cells *In vitro* in the presence of cisplatin results in cisplatin-resistant cells (HepG2/DDP) [10]. Cisplatin-resistance could be due to genomic alternations in certain gene(s) or/and rewiring of cellular signaling pathway(s) [11]. The detailed mechanism, however, remains poorly understood. Cisplatin has been shown to cause many side effects including nephrotoxicity, especially in aged population [12], therefore, efforts looking into the drug resistance mechanism are urgently needed.

The transcriptional factor nuclear factor erythroid-2 related factor 2 (NRF2) is a cap 'n' collar basic leucine zipper transcription factor which binds specifically to the antioxidant response element (ARE) at the promoter of its target genes [4, 13]. Nrf2 regulates a broad spectrum of genes involved in redox homeostasis, for example, NADP/ NADPH quinone oxidoreductase 1 (NQO1), heme oxygenase-1 (HO-1) and glutathione glutamate-cysteine ligases (GCLM and GCLC) [14, 15]. Under normal growing conditions, Nrf2 interacts with KEAP1 protein in the cytoplasm, which ensures Nrf2 posttranslational degradation through the ubiquitin-proteasome system (UPS). With stress, including metabolic stress such as imbalance of redox homeostasis, Nrf2 dissociates from KEAP1, enters cell nucleus and drives gene transcription to restore the cellular redox homeostasis.

Redox imbalance is a critical factor contributing to aging and age-relate disease, including liver cancer [16–18], Nrf2 has been shown to promote healthy aging in various model organisms [19, 20]. However, Nrf2 also promotes the survival of cancer cells treated with chemotherapeutic drugs [4, 15, 21–23]. Nrf2 is often hyperactive in cancers, especially liver cancers caused by environmental carcinogens [24]. Elevated expression of Nrf2 has been shown to correlate with differentiation, metastasis and growth of HCC [24] and serve as a negative prognosis factor for many cancers [24]. Cancer cells acquiring mutations on KEAP1 and Nrf2 genes are often drug resistance but the mechanisms remain poorly understood. There are studies showing that genes encoding the ATP-dependent drug efflux pumps MRP1 and MRP2 are directly regulated by Nrf2 pathway [25, 26]. Other studies show that Nrf2 confers drug resistance through systemic and more complex manners [4, 22].

Metformin is a well-known diabetes drug prescribed to type 2 diabetes (T2D) patients. Metformin has been shown to decrease hepatic glucose production, decrease intestinal absorption of glucose, and increase peripheral glucose uptake. However, the mechanisms at the molecular levels remain unclear [27]. Recently, several association studies reveal that metformin could have beneficial effects on preventing growth or relapse of cancers [28–32], including hepatocellular carcinoma [33]. Metformin has been shown to increase the sensitivity cancer cells to oxidative stress and therapeutic drugs [34–37], through AMP-activated protein kinase (AMPK) and the mechanistic target of rapamycin (mTOR) pathway [38, 39]. Recently, Nrf2 pathway has also been implicated in the beneficial effect of metformin on preventing chemoresistance [40–43]. However, the detailed mechanisms remain elusive.

In this study, by using the human liver cancer cell line HepG2, we investigated into the effect of metformin on cellular metabolism and gene transcription contributing to cisplatin resistance. Cisplatin is widely used to treat hepatocellular carcinoma [44] and other solid cancers including breast, testicular, and ovarian cancers [45]. We found that Nrf2 hyperactivation contributed to cisplatin resistance. Importantly, we found that metformin suppressed Nrf2 and decreased cisplatin resistance through enhanced glucose metabolisms and activation of glycolysis. Inhibition of glycolysis with 2-DG blocked metformin’s beneficial effect. To our knowledge, our study provides the first line of evidence demonstrating the importance of glycolysis in metformin regulation of chemosensitivity.

## RESULTS

### Nrf2 conferred cisplatin resistance in HepG2 hepatocellular carcinoma cells

**Figure 1:**
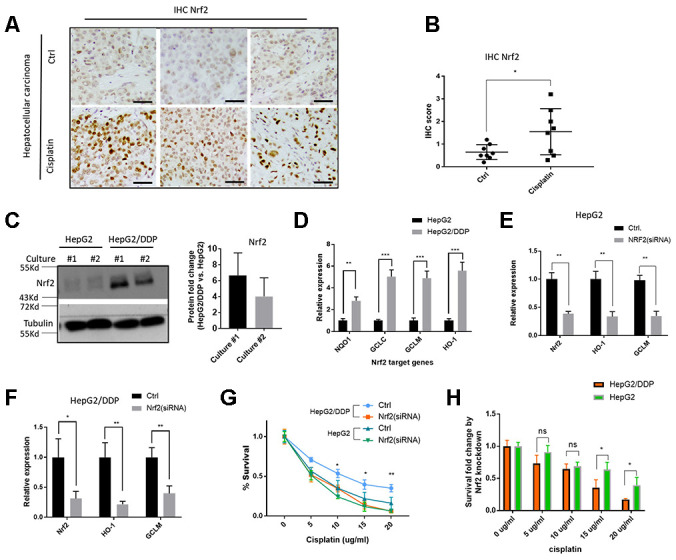
**Nrf2 activation contributes to cisplatin-resistance of HepG2/DDP cells.** (**A**) Nrf2 protein levels were increased in hepatocellular carcinoma tissues from patients undergone cisplatin treatment. Sections of paraffin-embedded hepatocellular carcinoma tissues were stained with a Nrf2-specific antibody in immunochemistry (IHC) experiments. Representative images were shown. Scale bars are 20 μm. (**B**) Quantifying Nrf2 expression in tumor tissues from 16 patients (8 undergone cisplatin treatment). Scores were obtained by pathologists according to hospital protocols. Statistical analysis by student’s t-test showed significant difference (*, P<0.05). (**C**) Nrf2 protein levels were elevated in cisplatin-resistant HepG2/DDP cells. Cell lysates of HepG2 and cisplatin-resistant HepG2/DDP from 2 different cultures were subjected to Western blot analysis with Nrf2 and Tubulin antibodies separately. Shown are samples from two different cultures. Representative images of were shown. Quantification of N= 2 biological repeats were shown in bar graph. (**D**) Nrf2 target gene expression was upregulated in HepG2/DDP cells. mRNA was isolated from HepG2 and HepG2/DDP and the relative mRNA levels of Nrf2 target genes (NQO1, GCLC, GCLM and HO-1) was compared by RT-qPCR. Relative expression of each gene as compared to that in HepG2 cells in fold change. Significance was tested by student’s t-test (** P<0.001, *** P<0.0001). (**E**, **F**) Nrf2 knockdown repressed target genes expression in HepG2 and HepG2/DDP cells. Nrf2 was knocked down by transfecting cells with siRNA pools specific to Nrf2 gene for 48 hours. mRNA was isolated and RT-qPCR was conducted with specific primers for both Nrf2 gene and its target genes (HO-1 and GCLM). For each gene, data were normalized to non-transfected controls (Ctrl). Significance was tested by student’s t-test (* P<0.05, ** P<0.001). (**G**) Nrf2 knockdown sensitized HepG2/DDP cells to cisplatin. HepG2/DDP and HepG2 cells were transfected with siRNAs specific to Nrf2 gene for 48 hours and cells were treated with cisplatin at indicated concentrations for 24 hours. Cell survival was measured with Cell Titer-Glo reagent. Data from 3 independent experiments was normalized to the average of non-treated controls. Significance was tested by student’s t-test (* P<0.05, ** P<0.001). (**H**) Survival fold change by Nrf2 knockdown. Data in (**G**) were used to calculate the fold change caused by Nrf2 siRNA knockdown at each cisplatin concentration. Significance was tested by student’s t-test (* P<0.05, ns, not significant).

Hyperactivation of the Nrf2 pathway has been reported to contribute to multiple drug resistance in some cancers [22, 23, 46, 47]. We tested if this was the case for hepatocellular carcinoma. To this end, we collected tissue samples from 16 patients (8 have undergone cisplatin treatment) with hepatocellular carcinoma and carried out immunohistochemistry (IHC) assay by using a specific Nrf2 antibody. The results showed that Nrf2 protein levels were increased in cisplatin-treated tumor tissues (Figure 1A and 1B). Second, we asked if Nrf2 was activated in cisplatin-resistant HepG2 cells (HepG2/DDP) as compared to parental HepG2 cells. The cisplatin-resistant HepG2/DDP cells were obtained by continuously culturing HepG2 cells in the presence of cisplatin, a process mimicking the clinical development of cisplatin resistance. Consistently, Western blot showed that HepG2/DDP cells expressed much higher levels of Nrf2 protein (Figure 1C). To further confirm the role of Nrf2, we carried out RT-qPCR experiment and found that HepG2/DDP cells had higher expression of Nrf2 target genes (NQO1, GCLC, GCLM and HO-1), compared with HepG2 (Figure 1D). Upon knocking down with Nrf2 siRNA, the expression of Nrf2 target genes was significantly reduced in both HepG2 and HepG2/DDP cells (Figure 1E and 1F). Last, we examined the cell viability through CellTiter-Glo, which detected intracellular ATP concentration proportional to the number of live cells. We found that siRNA knockdown of Nrf2 preferentially sensitized the HepG2/DDP cells to chemotherapy drug cisplatin (Figure 1G and 1H) at multiple concentrations, confirming the role of Nrf2 in the drug resistance of HepG2/DDP liver cancer cells. Consistently, activation of Nrf2 through knocking down the negative regulator KEAP1, preferentially increased cisplatin resistance in HepG2 cells compared to HepG/DDP cells (Supplementary Figure 1).

### Metformin sensitized HepG2/DDP cells to cisplatin through inhibiting Nrf2-dependent transcription

**Figure 2:**
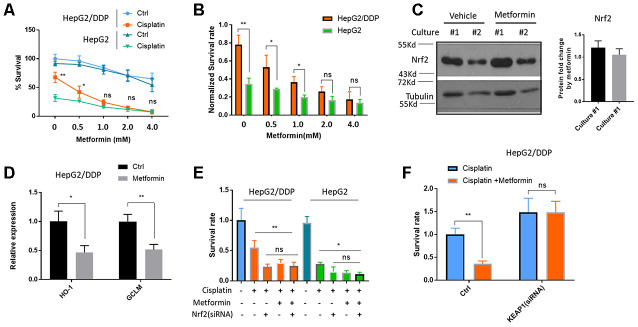
**Metformin increased cisplatin sensitivity of HepG2/DDP through down-regulation of Nrf2-dependent transcription.** (**A**) Metformin sensitized HepG2/DDP cells to cisplatin. HepG2/DDP and HepG2 cells were treated with metformin at various concentrations and cell survival was measured by Cell Titer-Glo. Relative percentage of survival after 10 ug/mL cisplatin treatment for 24 hours was plotted. Difference between cisplatin-treated HepG2/DDP and HepG2 was tested by student’s t-test (** P<0.001). (**B**) Data from (**A**) were normalized to cisplatin non-treated controls and shown in bar plot. Metformin’s effect on HepG2/DDP and HepG2 was tested by student’s t-test (** P<0.001). (**C**) Metformin treatment did not affect Nrf2 protein levels. HepG2/DDP cells were treated with or without 1mM metformin for 24 hours and total cell lysates were subjected to Western blotting. Culture #1 and #2 were different cell clones. Representative images of were shown. Quantification of N= 4 biological repeats were shown in bar graph. (**D**) Metformin repressed Nrf2 target genes expression. HepG2/DDP cells were treated with or without 1mM metformin for 24 hours and mRNA was isolated and reversed transcribed. RT-qPCR were carried out with HO-1 and GCLM specific primers. For each gene, data of cisplatin-treated samples were normalized to that of non-treated controls. Significance was tested by student’s t-test (* P<0.05, ** P<0.001). (**E**) Metformin and Nrf2(siRNA) had no additive effect on increasing cisplatin toxicity. HepG2/DDP and HepG2 cells were transfected with Nrf2-specific siRNAs for 24 hours then treated metformin for 24 hours. 10ug/ml cisplatin were added for another 24 hours and relative cell viability was measured with Cell Titer-Glo. Data from 2 independent experiments were normalized to the average of non-treated controls. Significance was tested by student’s t-test (ns, not significant, * P<0.01, ** P<0.001). (**F**) Nrf2 activation prevented metformin from increasing cisplatin toxicity. HepG2/DDP cells were treated as in (**E**) except KEAP1-specific siRNA was transfected. Data from 4 independent experiments were normalized to the average of metformin non-treated control. Significance was tested by student’s t-test (ns, not significant, ** P<0.001).

There are several studies and clinical trials showing the benefits of metformin on reducing cancer incidence [28–32]. Some studies suggest that metformin could benefit patients through improving their response to chemotherapy. We aimed to study the mechanisms at the cellular and molecular levels. First, we tested if metformin would increase cisplatin toxicity on HepG2/DDP cells. After 48 hours of 10 ug/ml of cisplatin treatment, ~ 60% of HepG2/DDP cells remained alive. However, adding metformin from 0.5-4 mM significantly enhanced the killing effect of cisplatin (Figure 2A). Although metformin also enhanced cisplatin toxicity in HepG2 cells, its effect on HepG2/DDP cells were much stronger (Figure 2B).

We then asked if metformin would sensitize the HepG2/DDP cells to cisplatin through down-regulating Nrf2. To this end, we first tested if metformin would change Nrf2 protein levels. We treated HepG2/DDP cells with 1 mM metformin. However, no change was observed by Western blotting Nrf2 in the cell lysates (Figure 2C). Second, we examined the change of Nrf2 target gene expression by RT-qPCR. Interestingly, although the protein levels of Nrf2 were not affected, the expression of Nrf2 target genes HO-1 and GCLM were robustly reduced (Figure 2D). Third, we asked if metformin’s effect on cisplatin toxicity was additive to Nrf2 knockdown. If metformin improves the killing of cisplatin through Nrf2 pathway, Nrf2 knockdown and metformin should not be additive. By knocking down Nrf2 and treat cancer cells with 1mM metformin, we showed that there was no additive effect on cisplatin killing of HepG2/DDP cells (Figure 2E). Last, we tested if constitutively activating Nrf2 would block metformin’s effect on enhancing cisplatin toxicity. Interestingly, KEAP1 knockdown promoted the survival of HepG2/DDP cells and prevented the beneficial effect of metformin (Figure 2F). Consistently, KEAP1 knockdown also blocked the effect of metformin on reducing Nrf2 target gene expression induced by cisplatin (Supplementary Figure 2). Together, our data suggest that metformin sensitizes HepG2/DDP cells to cisplatin through inhibiting Nrf2-dependent transcription.

### Metformin increased glucose uptake and glycolysis in HepG2/DDP cells

**Figure 3:**
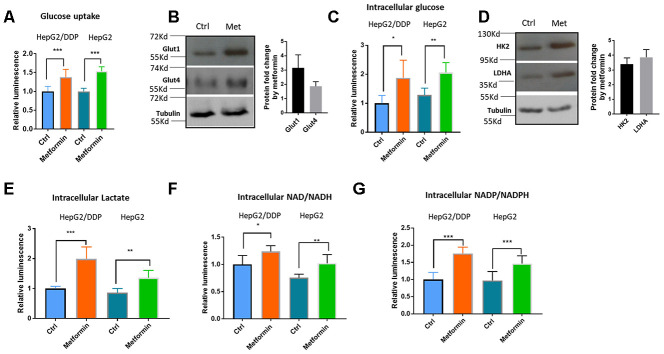
**Metformin increased glucose uptake and glycolysis in HepG2/DDP cells.** (**A**) Metformin increased glucose uptake. Indicated cells were treated with or without metformin (1mM) for 24 hours and glucose uptake assay were conducted with Glucose Uptake-Glo. Cell Titer-Glo was also carried out to measure the relative viability, which was used to normalize the data in glucose uptake assay. Data from 3 independent biological samples of 3 replicates were statistically analyzed by student’s t-test (*** P<0.0001). (**B**) Metformin increased the expression of glucose transporter Glut1 and Glut4. HepG2/DDP cells were treated with or without metformin (1mM) for 24 hours and total cell lysates were separated by SDS-PAGE. Glut1 and Glut4 protein levels were detected by Western blot using specific antibodies to Glut1 and Glut4. Tubulin was used as internal control. Representative images of were shown. Quantification of N= 2 biological repeats were shown in bar graph. (**C**) Metformin increased intracellular glucose concentration in HepG2/DDP cells. HepG2/DDP cells were treated with or without metformin (1mM) for 24 hours, washed extensively and intracellular glucose concentration was measured by using Glucose-Glo kit. Data from 2 independent biological samples of 3 replicates were plotted and statistically analyzed by student’s t-test (** P<0.001). (**D**) Metformin increased the protein levels of glycolytic enzymes HK2 and LDHA. Experiment was conducted as in (B) except HK2 and LDHA antibodies were used. Representative images of were shown. Quantification of N= 3 biological repeats were shown in bar graph. (**E**) Metformin increased intracellular lactate production. Indicated cells were treated with or without metformin (1mM) for 24 hours, washed extensively then intracellular lactate concentration was measured by using lactate-Glo kit. Data from 2 independent biological samples of 3 replicates were plotted and statistically analyzed by student’s t-test (** P<0.001, *** P<0.0001). (**F**) Metformin increased intracellular NAD/NADH production. HepG2/DDP cells were treated with or without metformin (1mM) for 24 hours and lactate concentration was measured by using NAD/NADH -Glo kit. Data from 2 independent biological samples of 3 replicates plotted and statistically analyzed by student’s t-test (* P<0.05, **P<0.001). (**G**) Metformin increased intracellular NADP/NADPH production. HepG2/DDP cells were treated with or without metformin (1mM) for 24 hours and lactate concentration was measured by using NADP/NADPH -Glo kit. Data from 2 independent biological samples of 3 replicates were plotted and statistically analyzed by student’s t-test (*** P<0.0001).

Metformin is a diabetes drug functioning to decrease plasma glucose levels. One of the possible mechanisms of such effect is through increasing glucose uptake in peripheral tissues such as fat tissues and muscles [27]. Also, several recent reports suggest that enhanced glucose metabolisms could down-regulate Nrf2 target genes expression [17, 19, 48]. We wondered if metformin would regulate Nrf2 transcriptional activity through modulating intracellular glucose metabolism. To this end, we first examined glucose absorption in HepG2/DDP cells after metformin treatment. Consistent with previous reports in muscle and fat cells [49–51], metformin increased glucose uptake in HepG2 cells, as determined by Glucose Uptake-Glo assay (Figure 3A). This was further confirmed by the upregulation of glucose transporter Glut1 and Glut4 as demonstrated by western blotting (Figure 3B). Knocking down GLUT1 gene preferentially reduced glucose uptake in HepG2/DDP cells compared to HepG2 (Supplementary Figure 3). Next, we asked if metformin could increase intracellular glucose levels in HepG2/DDP and HepG2 cells by using the Glucose-Glo Assay. Consistently, enhanced glucose uptake by metformin resulted in elevated intracellular glucose levels (Figure 3C). Third, we examined the protein levels of key enzymes in the glycolysis pathway, including the Hexokinase 2 (HK2) and Lactate dehydrogenase A (LDHA). Our results showed that these enzymes were elevated ~3 to 4 folds upon 1mM metformin treatment (Figure 3D). Last, we examined if increased glucose levels and glycolysis enzymes could result in elevated production of glycolytic metabolites such as lactate, NAD/NADH and NADP/NADPH. HepG2/DDP and HepG2 cells treated with and without metformin for 24 hours were trypsinized from plate and equal number of cells were used in the assay. As shown in Figure 3E–3G, lactate concentration was increased in metformin-treated cells, so were NAD/NADH and NADP/ NADPH levels. Metformin also increased the metabolite concentrations in a similar manner in HepG2 cells. Our results suggest that metformin increased glucose concentration and enhanced glycolytic activity in HepG2/DDP cells.

### High glucose suppressed Nrf2-mediated transcription and sensitized HepG2/DDP cells to cisplatin

**Figure 4:**
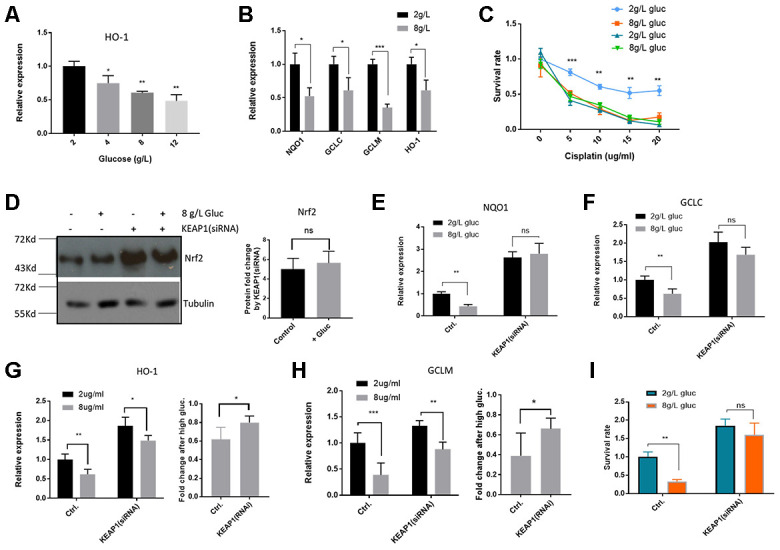
**High glucose medium suppressed Nrf2-mediated transcription in HepG2/DDP cells.** (**A**) High glucose decreased Nrf2 target gene HO-1 expression in a dose-dependent manner. HepG2/DDP cells were cultured RPMI-1640 with indicated concentrations of glucose medium for 24 hours and total mRNA was extracted and reverse transcribed. RT-qPCR was carried out to compare the relative expression of HO-1. Significance was tested by student’s t-test (*P<0.05, **P<0.001). (**B**) Glucose at 8g/L suppressed other Nrf2 target genes. Experiments were conducted as in (**A**) by using qPCR primers specific to NQO1, GCLC and GCLM. Data were normalized to 2g/L glucose (normal RPMI glucose concentration) for each individual gene. Significance was tested by student’s t-test (*P<0.05, ***P<0.0001). (**C**) Glucose at 8g/L increased cisplatin killing of HepG2/DDP cells. HepG2/DDP and HepG2 cells cultured in normal RPMI-1640) were shifted to normal (2g/L) or high glucose (8g/L) RPMI-1640 for 24 hours then cisplatin at indicated concentrations was added. After 24 hours, cell viability was measured with Cell Titer-Glo. Data from 2 independent experiments were normalized to the average of non-treated controls. Significance was tested by student’s t-test (** P<0.001, *** P<0.0001). (**D**) KEAP1 knockdown increased Nrf2 protein levels in a glucose-independent manner. HepG2/DDP cells were transfected with KEAP1-specific siRNAs for 24 hours and shifted to normal or high glucose medium for 24 hours. Total cell lysates were analyzed by SDS-PAGE and Western blotting. Representative images were shown. Quantification of N= 2 biological repeats were shown in bar graph. (**E**–**H**) KEAP1 knockdown prevented glucose from suppressing Nrf2 target genes. siRNA knockdown and glucose conditioning conducted as in (**D**). Relative expression of indicated genes was quantified by RT-qPCR. Comparison of fold change was shown in (**G**) and (**H**). Significance was tested by student’s t-test ((* P<0.05, ** P<0.001, ns, not significant). (**I**) KEAP1 knockdown preventing glucose from enhancing cisplatin toxicity. siRNA knockdown and glucose conditioning conducted as in (**D**). HepG2/DDP cells then treated with cisplatin (10ug/ml) for 24 hours. Relative cell viability was measured by Cell Titer-Glo. Data from 2 independent of 3 replicates were normalized to the control and analyzed with student’s t-test (** P<0.001, ns, not significant).

If increased glycolysis by metformin suppresses Nrf2-dependent transcription and improves cisplatin killing of HepG2/DDP cells, adding extra glucose in the medium might also show the same benefits. To test this possibility, HepG2/DDP cultured overnight in standard RPMI medium (containing 2g/L glucose) were shifted to RPMI medium with 2g/L, 4g/L, 8g/L or 12g/L glucose overnight. We first examined if Nrf2-dependent transcription of HO-1 gene would be suppressed using RT-qPCR. As shown in Figure 4A, glucose significantly suppressed HO-1 gene expression in a dose-dependent manner. Similarly, at the concentration of 8g/L glucose, expression of other Nrf2 target genes NQO1, GCLC and GCLM were also significantly reduced (Figure 4B). Next, we tested if high glucose medium could sensitize HepG2/DDP cells to cisplatin as did the metformin. Indeed, cells cultured in high glucose medium (8g/L) became more vulnerable to killing by cisplatin (Figure 4C).

To ask if glucose regulation of cisplatin sensitivity in HepG2/DDP cells was dependent on Nrf2 function, we siRNA knocked down the expression of the Nrf2 inhibitor KEAP1. KEAP1 was successfully inhibited as Nrf2 proteins were elevated in Western blot assay (Figure 4D). Interestingly, although high glucose medium suppressed the Nrf2 target genes (Figure 4A and 4B), Nrf2 was not affected at the protein levels (Figure 4D), similar to the results in metformin treatment (Figure 2C). Upon KEAP1 knockdown, glucose was no longer able to suppress Nrf2 target genes (Figure 4E-H). Consistently, upon KEAP1 knockdown, glucose was no longer able to increase cisplatin toxicity to HepG2/DDP cells (Figure 4I). Our data suggest that high glucose, similar to metformin, sensitizes HepG2/DDP cells to cisplatin through KEAP1-Nrf2 pathway.

### Metformin decreased Nrf2-dependent transcription and increased cisplatin sensitivity through glycolysis

**Figure 5:**
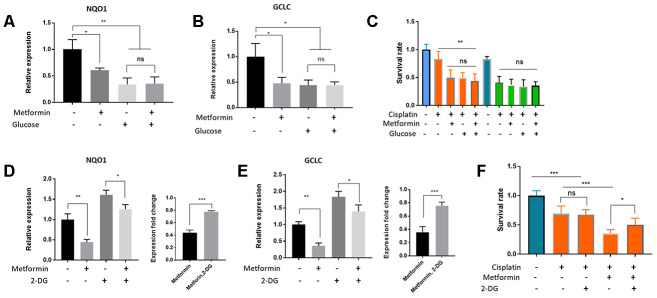
**Metformin regulated Nrf2-dependent transcription and cisplatin sensitivity through glucose metabolism and glycolysis in HepG2/DDP cells.** (**A**, **B**) Metformin and glucose had no additive effect on Nrf2-dependent transcription. HepG2/DDP cells in normal RPMI (2g/L) were shifted to normal or high glucose (8g/L) medium supplemented with or without 1mM metformin for 24 hours. Total mRNA was extracted and relative expression of indicated gene was quantified by RT-qPCR. Data from 2 independent experiments (3 replicates) were normalized to non-treated controls and statistically analyzed by student’s t-test (ns, not significant, *P<0.05, **P<0.001). (**C**) Metformin and glucose had no additive effect on cisplatin toxicity. HepG2/DDP cells cultured and treated with glucose and metformin as in (**B**). After cisplatin (10ug/ml) treatment for another 24 hours, relative cell viability was measured by Cell Titer-Glo. Data from 2 experiments with 3 replicates were plotted and analyzed by student’s t-test (** P<0.001, ns, not significant). (**D**–**E**) Inhibition of glycolysis with 2-DG (20mM) attenuated Metformin’s effect on repressing Nrf2-dependent target gene expression. HepG2/DDP cells under normal glucose condition were treated with or without 1mM metformin and 20mM 2-DG for 24 hours. Relative expression of indicated genes was quantified by RT-qPCR for 2 independent times (3 replicates each time). Right panels are normalized data to compare the effect of 2-DG. Student’s t-test was used to test statistical significance (*P<0.05, **P<0.001, ***P<0.0001). (**F**) Glycolysis inhibition by 2-DG attenuated metformin’s effect on increasing cisplatin toxicity. HepG2/DDP cells were cultured and treated with metformin as in (**E**) then cisplatin (10ug/ml) was added for 24 hours. Relative cell viability was measured by Cell Titer-Glo. Data from 2 experiments of 3 replicates were plotted and analyzed by student’s t-test (*P<0.05, ***P<0.0001, ns, not significant).

Metformin and high glucose both sensitize HepG2/DDP liver cancer cells to cisplatin (Figure 2A and Figure 4C). Whether they act through the same pathway in the upstream of Nrf2 remained unknown. To test this, we first asked if metformin and high glucose would act additively in suppressing Nrf2 target gene expression and cisplatin resistance of HepG2/DDP cells. Cell were cultured under normal and high glucose conditions and then treated with and without 1mM metformin. RT-qPCR showed that the target genes expression of NQO1 and GCLC were reduced by glucose or metformin treatment alone, but not further reduced by combining glucose and metformin, suggesting a non-additive effect (Figure 5A and 5B). Second, we treated HepG2/DDP cells cultured under above conditions with 10 ug/ml cisplatin and examined the viability. As shown in Figure 5C, glucose or metformin alone sensitized the HepG2/DDP cells to cisplatin toxicity, however, combination of metformin and glucose did not further increase cisplatin toxicity. These results suggest that high glucose and metformin target the same metabolic process (glycolysis) and pathway (Nrf2-dependent oxidative stress response pathway) to regulate drug response of HepG2/DDP cells.

Next, we chemically inhibited the cellular glycolysis by using 2-Deoxy-d-glucose (2-DG), a non-non-metabolizable glucose analog. 2-DG is a potent inhibitor of glycolysis via its action on hexokinase, the rate limiting step of glycolysis [52]. As shown in Figure 5D and 5E, metformin repressed Nrf2 target gene (NQO1 and GCLC) expression in both 2-DG treated and non-treated conditions. However, such repression was blunted by 2-DG (Figure 5D and 5E, right panels). Interestingly, 2-DG alone increased the expression of NQO1 and GCLC, consistent with a negative role of glycolysis in Nrf2 regulation. In addition, we tested the effect of 2-DG and metformin on cisplatin toxicity in HepG2/DDP cells. We found that the beneficial effect of metformin on enhancing cisplatin killing of HepG2/DDP cells was also significantly blunted by 2-DG (Figure 5F). Together, these data suggest that glycolysis is an important biological process mediating the effect of metformin and glucose on repressing Nrf2-dependent transcription and sensitizing HepG2/DDP cells to cisplatin.

## DISCUSSION

In recent years, there are great interests in repurposing the diabetes drug metformin for cancer treatment [28]. The idea is initially based on the observations that diabetes patients with cancer undergone metformin treatment show better prognosis, which was later confirmed by *in vitro* and *in vivo* studies in both animal models and clinical trials [28–33, 53]. The underlying mechanisms could involve increased drug sensitivity [34–37]. However, more studies are needed to understand the detailed mechanisms in order to accelerate the process of repurposing metformin for cancer treatment. The novel findings in the current study have given some insights into the mechanisms by which metformin repressed cisplatin resistance in hepatocellular carcinoma cells (HepG2/DDP). We found that metformin enhanced glucose metabolisms and glycolysis in HepG2/DDP cells, which in turn repressed Nrf2-dependent transcription, therefore contributing to cisplatin toxicity. Nrf2 is well known to cause drug resistance of many cancer cells [4, 15, 21–23]. Consistently, we showed that cisplatin-treated hepatocellular carcinomas expressed more Nrf2 proteins (Figure 1A and 1B), suggesting that Nrf2 activation is a pro-survival mechanism in response to chemotherapy. This is consistent with Nrf2’s role in extending lifespan and increasing pathogen resistance in simple model organisms [17, 19, 54]. Interestingly, although Nrf2-dependent transcriptions were down-regulated by metformin and high glucose, Nrf2 protein levels were not changed. This contrasts with widely reported mechanism, whereby Nrf2 was activated by escaping KEAP1-mediated posttranslational degradation through ubiquitin-proteasome system (UPS). Nrf2 isoforms have been reported in several types of cancer cells [55]. It is possible that the antibody did not detected the Nrf2 isoform regulated by glucose or metformin. Alternatively, Nrf2 could be regulated through different mechanisms. For example, there could be co-regulators of Nrf2 that transduce metformin and glucose signal to regulate Nrf2-dependent transcription through inhibiting/excluding Nrf2 interaction with transcription machinery. Interestingly, a recent study showed that Nrf2 homolog in *C. elegans* was regulated similarly as in our study [17], which could suggest a conserved and novel regulatory mechanism.

How does glycolysis regulate Nrf2 and cisplatin resistance? Although we found a robust effect of Nrf2 on promoting cisplatin resistance, the possibility cannot be ruled out that metformin, through glycolysis, modulates Nrf2-indpendent mechanisms to repress cisplatin-resistance of HepG2/DDP cells. However, our data at least demonstrate that Nrf2 is one of the contributors. The molecular connections between glycolysis and Nrf2 in term of cisplatin resistance awaits further investigations. Cancer cells generally prefer glycolysis for generating ATP [56], which in turn might slow down mitochondrial respiration, therefore reducing ROS production. As Nrf2 is responsive to ROS, it is possible that elevated glycolysis represses Nrf2 through lowering intracellular ROS levels. The pentose phosphate pathway (PPP) is a metabolic pathway parallel to glycolysis. Elevated glycolysis might also activate PPP to generate more NADPH, which in turn serve to reduce intracellular free radicals [57]. Less radicals could inactivate Nrf2 and contribute to cisplatin sensitivity. This is consistent with our observation that metformin increased NADPH and NADH production and repressed Nrf2-dependen transcription. In addition, emerging evidence show that glycolytic enzymes could function as signaling modulators in addition to their traditional roles [58]. These molecules could wire the signaling from glycolysis to Nrf2, contributing to cisplatin toxicity to HepG2 cells. However, further studies will be needed to either confirm or rule out these possible mechanisms.

The effect of hyperglycemia on chemo-resistance remains inconclusive [59]. There are quite a few studies showing a positive correlation of hyperglycemia with chemo-resistance in cancer patients. But *in vitro*, both positive and negative correlations have been reported and at least 5 cancer cell lines have been shown to be benefited from hyperglycemia [59]. For example, increased drug resistance is associated with reduced glucose levels [60]. Hyperglycemia increases toxicity of carboplatin and 5-fluorouracil in MCF-7 cells [61]. The positive correlation of blood glucose levels with chemo-resistance does not mean that intracellular glucose levels of cancer cells are increased. On the opposite, since a major reason causing hyperglycemia is impaired glucose uptake by peripheral tissues such as muscle, cancer cells in hyperglycemic patients could still have lower intracellular glucose, which could result in activation of Nrf2 and chemo-resistance. Alternatively, the opposing role of hyperglycemia on chemotherapy could be due to different mechanisms of drug resistance. For example, it is possible that only cancer cells having Nrf2 induction as a major drug resistance mechanism will be sensitive to high glucose; when other pathways dominate, glucose would exert a negative role on chemotherapy. These reasons could also explain the distinct observations in our current study as compared to some previous reports showing that metformin decreases glycolysis in many cancer cells.

Our results should not be extrapolated to animal or human studies without careful considerations, as genetic alterations and metabolic rewiring are inherent characteristics of cancer cells. For example, Nrf2 is well known to have dual roles in cancer progression, being able to promote cancer development and drug resistance on one hand but benefit chemotherapy on the other hand [62, 63]. Indeed, although Nrf2 was inhibited in some cancer cells in our study and other studies [37, 43, 64], there are several reports showing the opposite in other cells [65, 66]. Therefore, whether the role of metformin in glycolysis and chemoresistance function similarly *in vivo* awaits further investigation.

## MATERIALS AND METHODS

### Cell culture and drug treatment

RPMI-1640 medium containing 2g/L glucose was purchased from Sigma. HepG2 cells and HepG2/DDP cells were obtained from the Cell Bank, Chinese Academy of Sciences. HepG2 were maintained in RPMI-1640 supplemented with 5% fetal bovine serum. HepG2/DDP cells were maintained in the same medium with 0.1 ug/ml cisplatin. Metformin, Cisplatin, D-(+)-Glucose, 2-Deoxy-d-glucose (2-DG) were purchased from Sigma. Glucose were filter sterilized and add to RPMI-1640 (originally 2g/L glucose) to obtained high glucose medium (8g/L glucose). Other chemicals were added as indicated in the experimental results or figure legends.

### siRNA knockdown and RT-qPCR

*NRF2* and *KEAP1* knockdown was conducted as before [67]. Briefly, siRNAs (Supplementary Table 1) were mixed and added to basal media without FBS (Lonza, # CC-3131) with Hiperfect reagent (Qiagen) for 10 min at room temperature. The complex was then added to the cells cultured on 96-well plate (final siRNA pool concentration of 10 nM, 1 nM of each siRNA). Cells were then incubated for 48 hours in a humidified incubator. For RT-qPCR, cells were harvested and total RNA was isolated with trizol reagent and reverse transcribed with HiScript II Q RT SuperMix (Vazyme). qPCR was performed using AceQ Universal SYBR qPCR Master Mix (Vazyme) according to manufacturer’s protocol using primers shown in Supplemental Information, Supplementary Table 2. Actin gene was used as internal control.

### Cytotoxicity assay

Cytotoxicity of cisplatin was measured by using Celltiter-Glo from Promega according to manufacturer’s manual. Briefly, cells were cultured on 96-well plate and treated with cisplatin as indicated in each experiment. Medium were removed and 50 ul of PBS was added to the cells. 50 ul of Celltiter-glo reagent was added to the cells then cells were shaken at room temperature for 5 min on an orbital shaker. 50 ul of sample were transferred to opaque 384-well plate and the luminescent intensity were measured.

### Metabolites measurement

Glucose uptake was conducted by using Glucose Uptake-Glo from Promega. Briefly, Cells were cultured in 96-well plate, treated with 1mM metformin for 24 hours and washed 2 times with PBS. Samples were used for Glucose Uptake-Glo according to manufacturer’s protocol. Samples were prepared in triplicates and another setup was for cell viability assay to normalize the cell number. Relative intracellular glucose levels and lactate levels were measured with Glucose-Glo and lactate-Glo, respectively, from Promega according to manufacturer’s manual. Intracellular NAD/NADH and NADP/NADPH were measured by using NAD/NADH-Glo and NADP/NADPH-Glo from Promega. ATP assay was conducted by using Cell Titer-Glo from Promega, which is also used to normalized other metabolite concentrations.

### Immunohistochemistry (IHC)

Paraffin-embedded human hepatocellular carcinoma tissue slides were obtained from Shanghai Ninth People’s Hospital. More clinic information was provided in Supplementary Table 3. Slides were dewaxed by baking at 60 °C for 60 min then incubating for 10 min in xylene for 2 times under chemical hood. Xylene were removed by incubating slides in gradient ethanol (100%, 95%,70%) for 3 min each then rinsed with plenty of waters. Slides were then immersed in Antigen retrieval buffer (pH8.0) from Abcam and cooked in pressure rice cooker (temperature 105~110 °C) for 10 min to retrieve antigen. IHC was carried out by blocking with 5% goat serum for 15 min, incubated with anti-Nrf2 (Abcam, ab137550) at 400X for 60 min, washed extensively with PBS then incubated with secondary antibody (GeneTex One Step polymer-HRP) for 30 min. After rising with PBS, tissue samples were stained with Scytek DAB chromogen for 5 min, counterstained with hematoxylin for 1 min, clarified with 0.3% acid alcohol for 2~3 seconds, immediately rinsed in tap water. Slides were dehydrated by baking at 60 °C for about 5 min, immersed in xylene and covered with mounting medium and cover slide.

### Western blot

RPMI medium were removed from cells and cells attached to the 96-well plate were washed with PBS 1 time. 1X SDS-PAGE loading buffer (62.5 mM Tris-HCl pH 6.8; 2.5 % SDS; 0.002 % Bromophenol Blue; 0.7135 M (5%) β-mercaptoethanol; 10 % glycerol) were added directly to the cells. Cells were collected by pipetting and whole cell lysates were heated at 95 °C for 5 min. Whole lysates were subjected to SDS-PAGE and transferred to PVDF membrane. Membranes were blocked in 5% non-fat milk and probed with primary antibodies in 5% non-fat milk at the following concentration: Nrf2 (Promab Biotechnologies, #30597) at 2000X, Tubulin (Promab Biotechnologies, #20374) 0.2 ug/ml, Glut1 (R&D Systems, MAB14181) 2 ug/ml, Glut4 (Abcam, ab654) at 2500X dilution, HK2 (R&D Systems, MAB8179) at 0.2 ug/ml, LDHA (R&D Systems, AF7304). Membrane were washed with PBS extensively and probed with secondary antibodies (HRP-conjugated) at 10000X at 5% non-fat milk for 30 mins. Membrane was detected by enhanced chemiluminescence (ECL).

### Statistically analysis data quantification

Data shown in the bar graph were collected from at least 3 biological replicates if not otherwise indicated. Each biological replicate consists of at least 3 technical replicates. Data were pooled and analyzed by two tailed, paired student’s t-test in the GraphPad Prism software. Error bars stand for standard deviation of the mean (SD). Western blot signals were quantified through ImageJ.

## Supplementary Material

Supplementary Figures

Supplementary Tables
