# Supplementary material for: Metformin suppresses Nrf2-mediated chemoresistance in hepatocellular carcinoma cells by increasing glycolysis
Source: Aging (Albany NY). 2020 Sep 14;12(17):17582–600. doi: 10.18632/aging.103777 (PMC7521529; doi:10.18632/aging.103777)
Supplement: Supplementary Figures [file aging-12-103777-s002..pdf]

SUPPLEMENTARY FIGURES

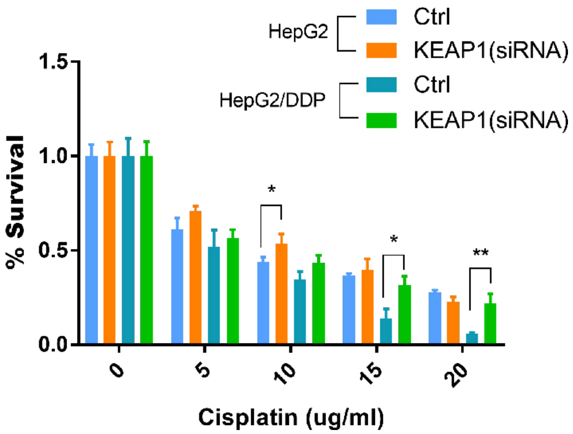

Supplementary Figure 1. Knockdown of KEAP-1 increases the resistance to cisplatin in HepG2 cells. HepG2 and HepG2/DDP cells were transfected with siRNAs specific to KEAP1 gene for 48 hours and cells were treated with cisplatin at indicated concentrations for 24 hours. Cell survival were measured with Cell Titer-Glo. Data were from at least 2 independent experiments and normalized to the average of non-treated controls. Significance were tested by student's t-test (* P<0.05, ** P<0.001).

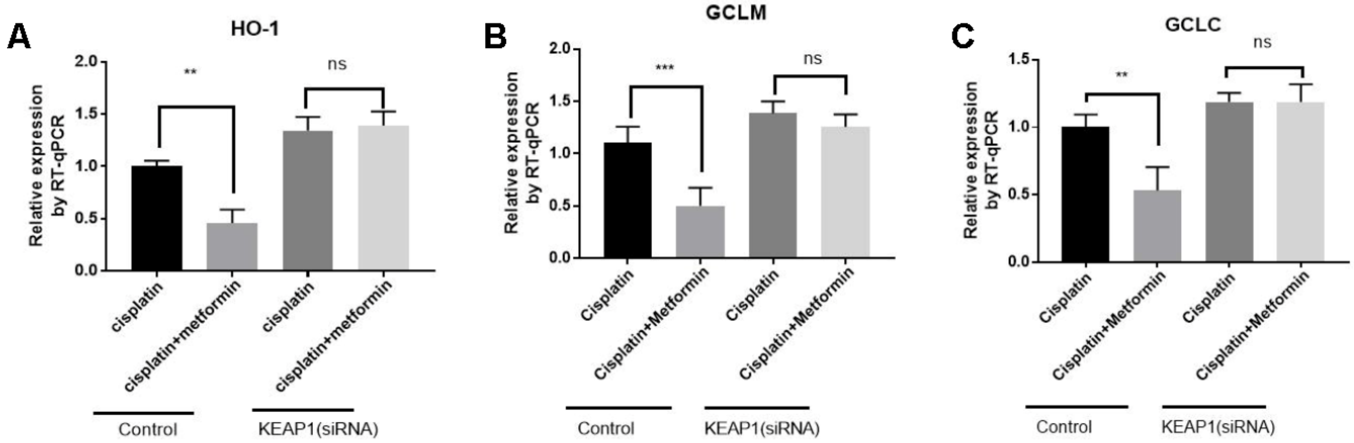

Supplementary Figure 2. KEAP1 knockdown prevents metformin from reducing Nrf1 target gene expression. RT-qPCR were used to compare the relative mRNA levels of the well-known Nrf2 target genes be: (NQO1 (**A**), GCLC (**B**) and GCLM (**C**)). Samples from 3 preparations were quantified by qPCR and normalized to the average of cisplatin-treated controls. Error bars stand for standard deviation. Groups were compared by two-tailed, paired student t-test (ns, not significant, ** P<0.001, ***P<0.0001).

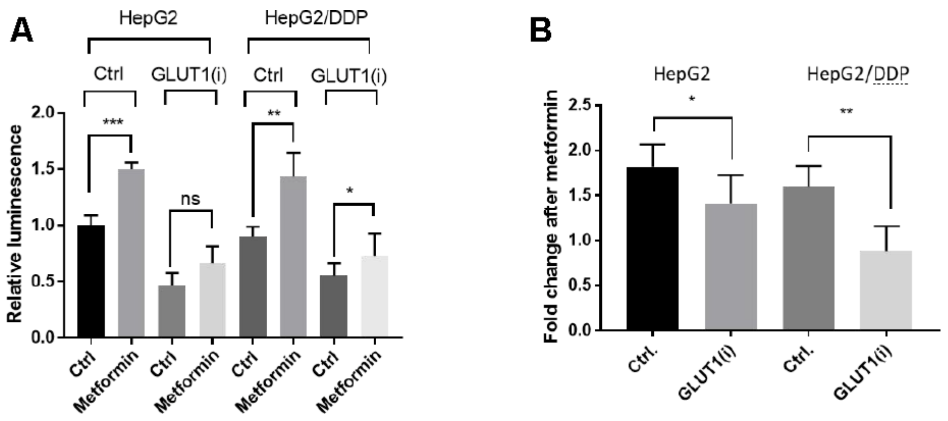

Supplementary Figure 3. GLUT1 knockdown blunts the effect of metformin on increasing glucose uptake. (**A**) GLUT1 knockdown effect on 2DG6P uptake. HepG2 and HepG2/DDP cells were first transfected with siRNA pools to knockdown GLUT1 glucose transporter for 48 hours, then treated with or without metformin (1mM) for 24 hours. Glucose uptake assay were conducted with Glucose Uptake-Glo by following manufacture (Promega) protocol. Glucose Uptake-Glo was also carried out to measure the relative glucose transport, which was used to normalize the data in glucose uptake assay. Data were from 3 independent biological samples of 3 replicates. Significance were tested by student's t-test (ns, not significant, ** P<0.001, *** P<0.0001). (**B**) Calculated fold changes were blunted by metformin treatment. Data of metformin treated groups for each sample were normalized to that of non-treated controls. Significance were tested by student's t-test (ns, not significant, * P<0.01, ** P<0.001).
