# Supplementary material for: Metformin suppresses Nrf2-mediated chemoresistance in hepatocellular carcinoma cells by increasing glycolysis
Source: Aging (Albany NY). 2020 Sep 14;12(17):17582–600. doi: 10.18632/aging.103777 (PMC7521529; doi:10.18632/aging.103777)
Supplement: Supplementary Tables [file aging-12-103777-s001..pdf]

## SUPPLEMENTARY TABLES

**Supplementary Table 1. siRNA pools used in this study.**

| | |
|---|---|
| NRF2 siRNA pool [1]: | |
| | NFE2L2-1 mGmAUUCUGACUCCGGCAUUUTT AmAAUGCCGGAGUCAGAAUCTT |
| | NFE2L2-2 mCmACUACUUGGCCUCAGUGATT UmCACUGAGGCCAAGUAGUGTT |
| | NFE2L2-3 mCmUCACAAGAGAUGAACUUATT UmAAGUUCAUCUCUUGUGAGTT |
| | NFE2L2-4 mGmAGAUGAACUUAGGGCAAATT UmUUGCCCUAAGUUCAUCUCTT |
| | NFE2L2-5 mGmCUCAUACUUUAUAAGUAATT UmUACUUAUAAAGUAUGAGCTT |
| | NFE2L2-6 mGmAGACUACCAUGGUUCCAATT UmUGGAACCAUGGUAGUCUCTT |
| | NFE2L2-7 mCmUGUUGAUUUAGACGGUAUTT AmUACCGUCUAAAUCAACAGTT |
| | NFE2L2-8 mGmCCCUCACCUGCUACUUUATT UmAAAGUAGCAGGUGAGGGCTT |
| | NFE2L2-9 mCmCAUUCACUCUCUGAACUUTT AmAGUUCAGAGAGUGAAUGGTT |
| | NFE2L2-10 mGmUCACUUGUUCCUGAUAUUTT AmAUAUCAGGAACAAGUGACTT |
| KEAP1 siRNA pool: | |
| | KEAP1-1 mCmGAAUGACAUCGGGCCGGATT UmCCGGCCCGAUGUCAUUCGTT |
| | KEAP1-2 mGmUGUUACGACCCAGAUACATT UmGUAUCUGGGUCGUAACACTT |
| | KEAP1-3 mCmCUUAAUUCAGCUGAGUGUTT AmCACUCAGCUGAAUUAAGGTT |
| | KEAP1-4 mCmAGAUUGGCUGUGUGGAGUTT AmCUCCACACAGCCAAUCUGTT |
| | KEAP1-5 mGmCUAUGAUGGUCACACGUUTT AmACGUGUGACCAUCAUAGCTT |
| | KEAP1-6 mGmGUUCUACGUCCAGGCGCUTT AmGCGCCUGGACGUAGAACCTT |
| | KEAP1-7 mGmCAUCAACUGGGUCAAGUATT UmACUUGACCCAGUUGAUGCTT |
| | KEAP1-8 mGmGGACAAACCGCCUUAAUUTT AmAUUAAGGCGGUUUGUCCCTT |
| | KEAP1-9 mCmGCCUUAAUUCAGCUGAGUTT AmCUCAGCUGAAUUAAGGCGTT |
| | KEAP1-10 mCmGAAUGAUCACAGCAAUGATT UmCAUUGCUGUGAUCAUUCGTT |
| GLUT1 siRNAs [2] | |
| | Sense 5'- CCUCUUUGUUAAUCGCUUU -3', Antisense: 5'- AAAGCGAUUAACAAAGAGG -3' |

**Supplementary Table 2. RT-qPCR primers for Nrf2 target genes.**

| | | |
|---|---|---|
| NRF2 | 5′-CAGCGACGGAAAGAGTATGA-3′ | 5′-TGGGCAACCTGGGAGTAG-3′ |
| Actin | 5′-CTGGAACGGTGAAGGTGACA-3′ | 5′-AAGGGACTTCCTGTAACAATGCA-3′ |
| GCLC | 5′-CCCTCGCTTCAGTACCTTAAC-3′ | 5′-GACAGCAATTGCCCATTCCA-3′ |
| GCLM | 5′-AGTGGGCACAGGTAAAACCA-3′ | 5′-CTCGTGCGCTTGAATGTCAG-3′ |
| HO-1 | 5′-CTGCTCAACATCCAGCTCTTTG-3′ | 5′-AGTGTAAGGACCCATCGGAGA-3′ |
| NQO1 | 5′-CAAAGGACCCTTCCGGAGTAA-3′ | 5′-ACTTGGAAGCCACAGAAATGC-3′ |

**Supplementary Table 3. Clinical information of patients in immunohistochemistry experiment in Figure 1A–1B.**

| Patent number | Age | Gender | Cancer stage | Surgery | Cisplatin treatment |
|---|---|---|---|---|---|
| 1 | 78 | M | IIIA | N | Y |
| 2 | 62 | M | IIIB | Y | Y |
| 3 | 83 | M | IIIB | N | Y |
| 4 | 74 | M | II | N | Y |
| 5 | 66 | F | IIIA | N | Y |
| 6 | 80 | F | IIIA | N | Y |
| 7 | 76 | F | IIIB | Y | Y |
| 8 | 47 | F | IIIA | Y | Y |
| 9 | 69 | M | IVA | N | N |
| 10 | 77 | M | IVA | N | N |
| 11 | 65 | M | IIIB | Y | N |
| 12 | 74 | M | IVA | N | N |
| 13 | 83 | F | IIIB | N | N |
| 14 | 73 | F | IVA | M | N |
| 15 | 56 | F | IVA | N | N |
| 16 | 75 | F | IIIB | N | N |

## SUPPLEMENTARY REFERENCES


1. Fourtounis J, Wang IM, Mathieu MC, Claveau D, Loo T, Jackson AL, Peters MA, Therien AG, Boie Y, Crackower MA. Gene expression profiling following NRF2 and KEAP1 siRNA knockdown in human lung fibroblasts identifies CCL11/Eotaxin-1 as a novel NRF2 regulated gene. Respir Res. 2012; 13:92. https://doi.org/10.1186/1465-9921-13-92 PMID:23061798

2. Ida-Yonemochi H, Nakatomi M, Harada H, Takata H, Baba O, Ohshima H. Glucose uptake mediated by glucose transporter 1 is essential for early tooth morphogenesis and size determination of murine molars. Dev Biol. 2012; 363:52–61. https://doi.org/10.1016/j.ydbio.2011.12.020 PMID:22226978
